# Effectiveness of Pneumococcal Conjugate Vaccines (PCV7 and PCV13) against Invasive Pneumococcal Disease among Children under Two Years of Age in Germany

**DOI:** 10.1371/journal.pone.0161257

**Published:** 2016-08-15

**Authors:** Mark van der Linden, Gerhard Falkenhorst, Stephanie Perniciaro, Christina Fitzner, Matthias Imöhl

**Affiliations:** 1 National Reference Center for Streptococci, Department of Medical Microbiology, University Hospital (RWTH), Aachen, Germany; 2 Department for Infectious Disease Epidemiology, Robert Koch Institute, Berlin, Germany; 3 Department of Medical Statistics, University Hospital (RWTH), Aachen, Germany; Universidade de Lisboa Faculdade de Medicina, PORTUGAL

## Abstract

**Background:**

In this study we calculate the effectiveness of pneumococcal conjugate vaccines (PCV) against invasive pneumococcal disease (IPD) among children under the age of two years using the indirect cohort method. We also discuss the timeliness of vaccination and the residual cases of vaccine type IPD.

**Methods and Findings:**

From July 2006 until June 2015, 921 IPD cases were reported and for 618 children (67.1%), the vaccination status at the time of infection could be accurately determined. Of these, 379 (61.3%) were vaccinated and 239 (38.7%) were not vaccinated. The adjusted vaccine effectiveness (VE) of PCV7 for all included serotypes + 6A was 80% (95% CI: 63–89) for at least one dose, 97% (89–100) after three primary doses (post primary) and 95% (57–100) post booster. The adjusted overall VE of PCV13 was 86% (74–93) for at least one dose, 85% (62–94) post primary and 91% (61–99) post booster. For the additional serotypes included in PCV13, the adjusted VE was 82% (66–91), 80% (46–93) and 90% (54–98) respectively. The serotype specific VE for at least one dose was high for serotypes 1 (83%; 15–97), 3 (74%; 2–93), 7F (84%; 18–98) and 19A (77%; 47–90). Only 39.5% of children with IPD obtained their first dose of PCV7 according to schedule (2^nd^ dose: 32.9%, 3^rd^ dose: 22.0%, booster dose: 63.6%). For children vaccinated with PCV13 values were slightly better: 43.8%, 33.5%, 26.3% and 74.3% respectively. Among 90 residual cases with PCV7 serotypes, 73 (81.1%) were in unvaccinated children, and 15 (16.7%) in children who had not obtained the number of doses recommended for their age, and only two (2.2%) in children vaccinated according to age. Of 82 cases with PCV13 serotypes occurring after the switch from PCV7 to PCV13, 56 (68.3%) were not vaccinated, 22 (26.8%) were incompletely vaccinated, and four (4.9%) were vaccinated according to age.

**Conclusions:**

Our data show a high effectiveness of pneumococcal conjugate vaccination in Germany. However, the administration of vaccine doses among children with IPD is often delayed, resulting in many vaccine type cases in non- or incompletely-vaccinated children. Whether the recently-implemented change to a 2+1 schedule will improve the timeliness of vaccination should be subject to careful monitoring.

## Introduction

*Streptococcus pneumoniae* remains a major cause of infectious disease, especially among the very young (children <5 years of age) and the elderly (>60 years of age). Apart from causing diseases like otitis media, pneumonia and meningitis, pneumococci colonize the nasopharynx, with the highest level of carriage reported from pre-school age children [1, 2]. Pneumococcal conjugate vaccines have been developed to reduce the burden of disease among children and, through mucosal immunity, also reduce the carriage of serotypes included in the vaccine.

The first pneumococcal conjugate vaccine, Prevenar (PCV7), comprising the serotypes 4, 6B, 9V, 14, 18C, 19F and 23F, became available in Germany in 2001. Initially, the German Standing Committee on Vaccination (STIKO) recommended vaccination of children with an increased risk for pneumococcal disease only. Universal vaccination of all children under two years of age was recommended in July 2006, with three primary doses at age 2, 3 and 4 months and a booster at age 11 to 14 months (3 + 1 schedule) [3]. Vaccination was performed with PCV7 until 2009 when higher valent pneumococcal conjugate vaccines became available. In April 2009, Synflorix (PCV10), including the PCV7 serotypes plus serotypes 1, 5 and 7F, was licensed, and in December 2009, Prevenar13 (PCV13), including the PCV10 serotypes plus serotypes 3, 6A and 19A, replaced PCV7 in Germany. Vaccination costs are fully reimbursed by the German health insurance companies. The choice of vaccine formulation lies with the parents and the pediatrician. Currently, the vast majority of children are vaccinated with PCV13 [4]. The uptake of pneumococcal conjugate vaccine among children under two years of age in Germany is 80–85% (as based on sold/prescribed doses) [5–8]. In Germany, the introduction of childhood pneumococcal conjugate vaccination has had a profound effect on invasive pneumococcal disease (IPD) and otitis media among children, as well as on the serotype distribution of IPD among adults [4, 9].

In this study, we calculate the vaccine effectiveness (VE) of PCV7 and PCV13 against IPD among children under the age of two years using the indirect cohort method described by Broome [10]. We also report on the timeliness of pneumococcal conjugate vaccination and on the residual cases with vaccine type IPD.

## Materials and Methods

### Data collection

Surveillance of IPD among children in Germany has been conducted by the German National Reference Center for Streptococci (GNRCS) since 1997 and the surveillance system has been described previously [4]. In this study only cases sent in from July 2006, when childhood pneumococcal conjugate vaccination was recommended, were used. IPD cases were defined as *Streptococcus pneumoniae* isolated from blood, cerebrospinal fluid or any other normally sterile body fluid. A questionnaire requesting data on age, gender, diagnosis and vaccination status (type of vaccine, vaccination date, batch number) was filled out by the diagnostic laboratories and sent in together with the pneumococcal isolates. Incomplete data on vaccination status were ascertained by contacting the laboratories and treating pediatricians. All data were entered into the database of the GNRCS in an anonymized way. Cases were grouped per pneumococcal season (from July to June of consecutive years). Since the vaccination status was ascertained retrospectively, all data were processed to reflect the vaccination status of each child at the time of infection, i.e. all vaccination doses given after the time of infection were disregarded. To assess the timeliness of pneumococcal conjugate vaccination in Germany, the actual age at vaccination was compared to the scheduled age (first dose: 60–89 days of age, second dose: 90–119 days, third dose: 120–149 days, booster: 330–449 days). The analysis was performed for PCV7 and PCV13.

### Characterization of isolates and serotyping

Species identification was performed at the GNRCS using bile and optochin testing. In dubious cases, PCR analysis of several genes was performed (*ply*, *lytA*, *sodA*, 16S-rRNA). As a last resort, MLST was performed. Pneumococcal isolates were serotyped by Neufeld’s Quellung reaction using type and factor sera provided by the Statens Serum Institut, Copenhagen, Denmark. Isolates were considered non-typeable when there was no reaction with any of the antisera.

### Calculation of vaccine effectiveness

VE was calculated using the indirect cohort method as described by Broome [10]. In this calculation, patients with non-vaccine serotype IPD are used as controls for the cases of patients with vaccine serotype IPD. The assumption is that the risk of developing non-vaccine serotype disease is independent of the patient’s vaccination status. A comparison of the odds of vaccination in patients with vaccine type and non-vaccine type disease allows for the calculation of VE. VE was calculated for children having obtained one or more doses of vaccine (at least one dose), for children with a completed primary schedule, i.e. three doses and age <449 days at onset of disease (post primary) and for children with a completed schedule, i.e. four doses (post booster).

A vaccination dose was only counted when given at least fourteen days before onset of disease. Children aged <74 days at onset of disease, i.e. too young to be vaccinated, were excluded from the analysis. The maximum age for inclusion in the study was 729 days at onset of disease. The following age cohorts were used for VE calculation: children aged 74–729 days were included in the ‘at least one dose’ analysis, children aged 150-<449 days in the ‘post primary’ cohort and children aged 330–729 days in the ‘post booster’ cohort.

Only children with complete information on vaccination status, i.e. date of vaccination and type of vaccine, were included in the analysis. To reflect the conjugate vaccine introduction in Germany, the analysis was split in two periods. From July 2006 until June 2010, children vaccinated with PCV7 were analysed. From July 2010 until June 2015, vaccinations with PCV13 were assessed. Due to the very low use of PCV10 in Germany, and the resulting low number of cases of PCV10-vaccinated children, a calculation of PCV10 VE was not performed.

### Statistical analysis

Vaccine effectiveness was calculated using the formula: VE = 1 –odds of being vaccinated in a vaccine type case / odds of being vaccinated in a non-vaccine type case. Odds ratios (OR) and 95% confidence intervals (CI) were estimated using Firth's bias-reduced logistic regression as described by Heinze [11]. Confidence intervals that do not cross zero are considered statistically significant. VE was adjusted for age and season following the issue of the vaccination recommendation. All calculations were performed using R (version 3.1.1, The R Foundation for Statistical Computing, Vienna, Austria).

### Ethical statement

An ethical approval was not required since the study was performed with *Streptococcus pneumoniae* isolates that resulted from routine microbiological diagnostic procedures as requested by the treating physician. No additional biological specimens were taken for the purpose of this study. Specimens were anonymized and only data on birthdate, sex, type of specimen, vaccination status and hospital/laboratory where the case was diagnosed were registered.

## Results

From July 2006 until June 2015, a total of 921 cases of IPD among children under two years of age were reported to the GNRCS. Ages varied from 0 to 23 months with 244 (26.5%) children aged 0–5 months, 282 (30.6%) aged 6–11 months, 249 (27.0%) aged 12–17 months and 146 (15.9%) aged 18–23 months. 552 (59.9%) patients were male, 357 (38.8%) female and for 12 patients gender was not reported.

**Fig 1:**
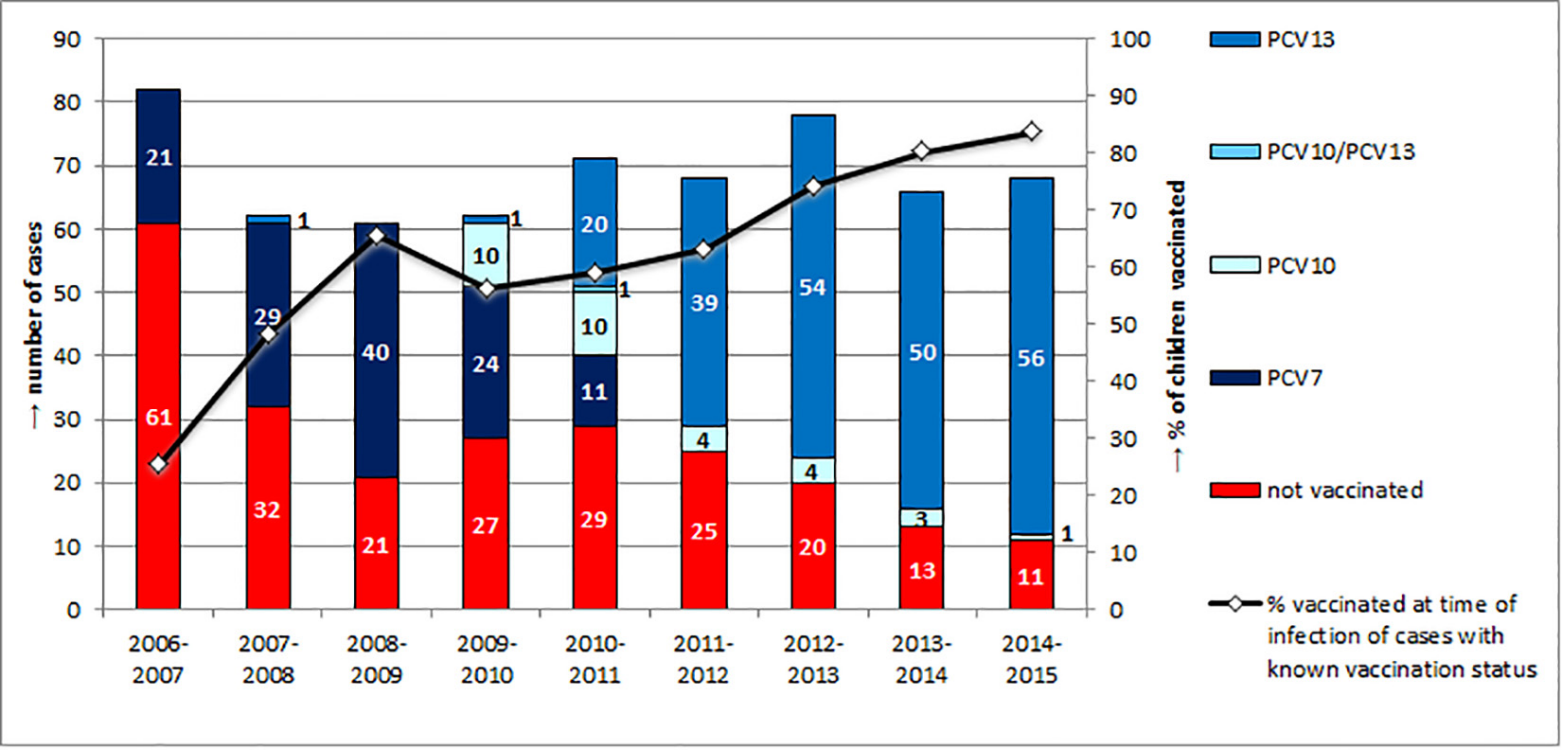
Vaccination status at time of infection of 618 children with IPD reported to the GNRCS from July 2006 –June 2015. Red: not vaccinated, dark blue: vaccinated with PCV7, light blue: vaccinated with PCV10, blue: vaccinated with PCV13. The black line represents the percentage of children vaccinated at the time of infection among the cases for which the vaccination status could be ascertained. Left Y-axis: number of cases, right y-axis: percentage of vaccinated children.

For 165 (17.9%) children the vaccination status could not be ascertained, for 63 (6.8%) children vaccination data were incomplete and 75 (8.1%) children were excluded from the analysis because they were younger than 74 days. For 618 children (67.1%), the vaccination status at the time of infection could be accurately determined. Of these, 379 (61.3%) were vaccinated and 239 (38.7%) were not vaccinated. Among the vaccinated children, 125 (33.0%) were vaccinated with PCV7, 32 (8.4%) with PCV10 and 221 (58.3%) were vaccinated with PCV13 at the time of infection. One child had received a primary series of PCV10 and a booster with PCV13 and this case was excluded from further analysis. The percentage of children vaccinated with at least one dose of any PCV at the time of infection gradually increased from 25.6% in 2006–2007 to 83.8% in 2014–2015 (**Fig 1**).

Serotype distribution varied over time as a result of the vaccination with pneumococcal conjugate vaccines. Both during the PCV7 period as well as during the PCV13 period the number of IPD cases caused by serotypes included in the respective vaccines decreased every year. [4]

### Vaccine effectiveness

For the analysis of PCV7 vaccine effectiveness, 255 IPD cases fulfilling the above described criteria (age 74–729 days, case between July 2006 and June 2010) were included. Ten cases vaccinated with PCV10 and two cases vaccinated with PCV13 (most of them reported in 2009–2010) were excluded (**Fig 1**). The adjusted VE for all PCV7 serotypes including 6A was 80% for at least one dose, 97% post primary and 95% post booster (**Table 1**). The adjusted VE for single serotypes included in PCV7 in the ‘at least one dose’ cohort varied from 8% for serotype 18C to 90% for serotypes 6B and 14, for post primary VEs varied from 51% for serotype 4 to 97% for serotype 6B and 14, for post booster most VE values were lower and with wide CIs crossing zero, due to low numbers of both vaccinated and unvaccinated cases. For serotype 6A, adjusted VEs of 73% for at least one dose, 91% post primary and 74% post booster were calculated which is consistent with cross-protection of serotype 6B antibodies induced by PCV7 towards this serotype. However, the VE values had wide CIs, and the cohort sizes of the latter two age groups had very low numbers.

**Table 1:** Vaccine Effectiveness of PCV7 among children under two years of age with IPD in Germany, July 2006 till June 2010. Values in bold are statistically significant.

2006–2010		cases vaccinated: unvaccinated	controls vaccinated: unvaccinated	crude vaccine effectiveness (95% CI)	adjusted vaccine effectiveness (95% CI)
PCV7 serotypes + 6A*					
** **	**at least one dose**	20:81	94:60	**84% (72 to 91)**	**80% (63 to 89)**
** **	**post primary**	1:49	38:27	**98% (91 to 100)**	**97% (89 to 100)**
** **	**post booster**	0:44	11:19	**98% (84 to 100)**	**95% (57 to 100)**
**Serotype 4**					
** **	**at least one dose**	0:1	94:60	79% (-307 to 100)	70% (-636 to 100)
** **	**post primary**	0:1	38:27	76% (-364 to 100)	51% (-1088 to 100)
** **	**post booster**	0:1	11:19	44% (-1054 to 100)	-86% (-50978 to 99) ^¶^
**Serotype 6B**					
** **	**at least one dose**	2:20	94:60	**92% (74 to 98)**	**90% (66 to 98)**
** **	**post primary**	0:15	38:27	**98% (82 to 100)**	**97% (72 to 100)**
** **	**post booster**	0:14	11:19	94% (48 to 100)	85% (-89 to 100)
**Serotype 9V**					
** **	**at least one dose**	0:4	94:60	93% (32 to 100)	89% (-13 to 100)
** **	**post primary**	0:2	38:27	86% (-85 to 100)	83% (-158 to 100)
** **	**post booster**	0:2	11:19	66% (-367 to 100)	-16% (-18054 to 99) ^¶^
**Serotype 14**					
** **	**at least one dose**	2:24	94:60	**93% (79 to 99)**	**90% (68 to 98)**
** **	**post primary**	0:12	38:27	**97% (77 to 100)**	**97% (71 to 100)**
** **	**post booster**	0:15	11:19	**95% (52 to 100)**	89% (-20 to 100)
**Serotype 18C**					
** **	**at least one dose**	4:6	94:60	56% (-52 to 88)	8% (-239 to 76)
** **	**post primary**	0:3	38:27	90% (-11 to 100)	79% (-137 to 100)
** **	**post booster**	0:4	11:19	81% (-103 to 100)	66% (-635 to 100)
**Serotype 19F**					
** **	**at least one dose**	5:11	94:60	69% (14 to 90)	55% (-34 to 87)
** **	**post primary**	1:6	38:27	84% (15 to 98)	73% (-44 to 97)
** **	**post booster**	0:4	11:19	81% (-103 to 100)	72% (-301 to 100)
**Serotype 23F**					
** **	**at least one dose**	3:8	94:60	**74% (11 to 94)**	61% (-62 to 92)
** **	**post primary**	0:6	38:27	95% (50 to 100)	89% (-23 to 100)
** **	**post booster**	0:3	11:19	76% (-184 to 100)	33% (-9761to 100)
**Serotype 6A**					
** **	**at least one dose**	4:7	94:60	62% (-26 to 90)	73% (1 to 93)
** **	**post primary**	0:4	38:27	92% (21 to 100)	91% (8 to 100)
** **	**post booster**	0:1	11:19	43% (-1054 to 100)	74% (-478 to 100)

* Serotype 6A is not included in PCV7, but antibodies against serotype 6B are crossprotective towards 6A.

^¶^This value can not be strictly interpreted due to insufficient number of events

The VE for PCV13 was calculated for the period July 2010 till June 2015 and included 317 cases (age 74–729 days, and excluding 11 cases vaccinated with PCV7 and 22 cases vaccinated with PCV10 and most of them reported in 2010–2011 (**Fig 1**). For all PCV13 serotypes, the adjusted VE was 86% for at least one dose, 85% post primary and 91% post booster (**Table 2**). For the six serotypes unique to PCV13 (PCV13-non-PCV7 serotypes), adjusted VE values were 82%, 80% and 90% respectively. For the 7 serotypes present in both PCV7 and PCV13, adjusted VEs were 94%, 99% and 83% respectively. Serotype specific VE was high for serotypes 1, 6A, 7F and 19A. Serotype 5 cases are very rare in Germany and did not occur in the PCV13 analysis period. For serotype 3, the VE for ‘at least one dose’ was 74%. VEs for ‘post primary’ and ‘post booster’ were 80% and 63% respectively, however, with wide 95% CIs and lower limits below zero and very small cohort sizes.

**Table 2:** Vaccine Effectiveness of PCV13 among children under two years of age with IPD in Germany, July 2010 till June 2015. Values in bold are statistically significant.

2010–2015		cases vaccinated: unvaccinated	controls vaccinated: unvaccinated	crude vaccine effectiveness (95% CI)	adjusted vaccine effectiveness (95% CI)
**PCV13 serotypes**					
** **	**at least one dose**	25:55	194:43	**90% (82 to 94)**	**86% (74 to 93)**
** **	**post primary**	10:22	74:20	**87% (70 to 95)**	**85% (62 to 94)**
** **	**post booster**	2:13	33:16	**91% (65 to 98)**	**91% (61 to 99)**
**PCV13-non-PCV7 serotypes**					
** **	**at least one dose**	23:43	194:43	**88% (78 to 93)**	**82% (66 to 91)**
** **	**post primary**	10:16	74:20	**82% (57 to 93)**	**80% (46 to 93)**
** **	**post booster**	2:12	33:16	**90% (62 to 98)**	**90% (54 to 98)**
**PCV7 serotypes in PCV13**					
** **	**at least one dose**	2:12	194:43	**96% (84 to 99)**	**94% (78 to 99)**
** **	**post primary**	0:6	74:20	98% (81 to 100)	99% (80 to 100)
** **	**post booster**	0:1	33:16	84% (-225 to 100)	83% (-240 to 100)
**Serotype 1**					
** **	**at least one dose**	2:5	194:43	90% (56 to 98)	83% (15 to 97)
** **	**post primary**	1:2	74:20	84% (-31 to 99)	49% (-614 to 96)
** **	**post booster**	0:1	33:16	84% (-225 to 100)	82% (-328 to 100)
**Serotype 3**					
** **	**at least one dose**	6:5	194:43	74% (10 to 92)	74% (2 to 93)
** **	**post primary**	1:2	74:20	84% (-31 to 99)	80% (-68 to 98)
** **	**post booster**	1:2	33:16	70% (-140 to 97)	63% (-393 to 97)
**Serotype 5**					
** **	**at least one dose**	0:0	194:43		
** **	**post primary**	0:0	74:20		
** **	**post booster**	0:0	33:16		
**Serotype 6A**					
** **	**at least one dose**	0:4	194:43	95% (55 to 100)	96% (56 to 100)
** **	**post primary**	0:1	74:20	91% (-78 to 100)	84% (-214 to 100)
** **	**post booster**	0:2	33:16	90% (-30 to 100)	84% (-224 to 100)
**Serotype 7F**					
** **	**at least one dose**	1:12	194:43	**97% (88 to 100)**	**84% (18 to 98)**
** **	**post primary**	0:2	74:20	95% (29 to 100)	86% (-116 to 100)
** **	**post booster**	0:0	33:16	51% (-9185 to 100)	32% (-8066 to 99)
**Serotype 19A**					
** **	**at least one dose**	14:17	194:43	**81% (60 to 92)**	**77% (47 to 90)**
** **	**post primary**	8:9	74:20	**75% (30 to 92)**	**73% (18 to 92)**
** **	**post booster**	1:7	33:16	**90% (49 to 99)**	**88% (25 to 99)**

### Timeliness of vaccination

**Fig 2:**
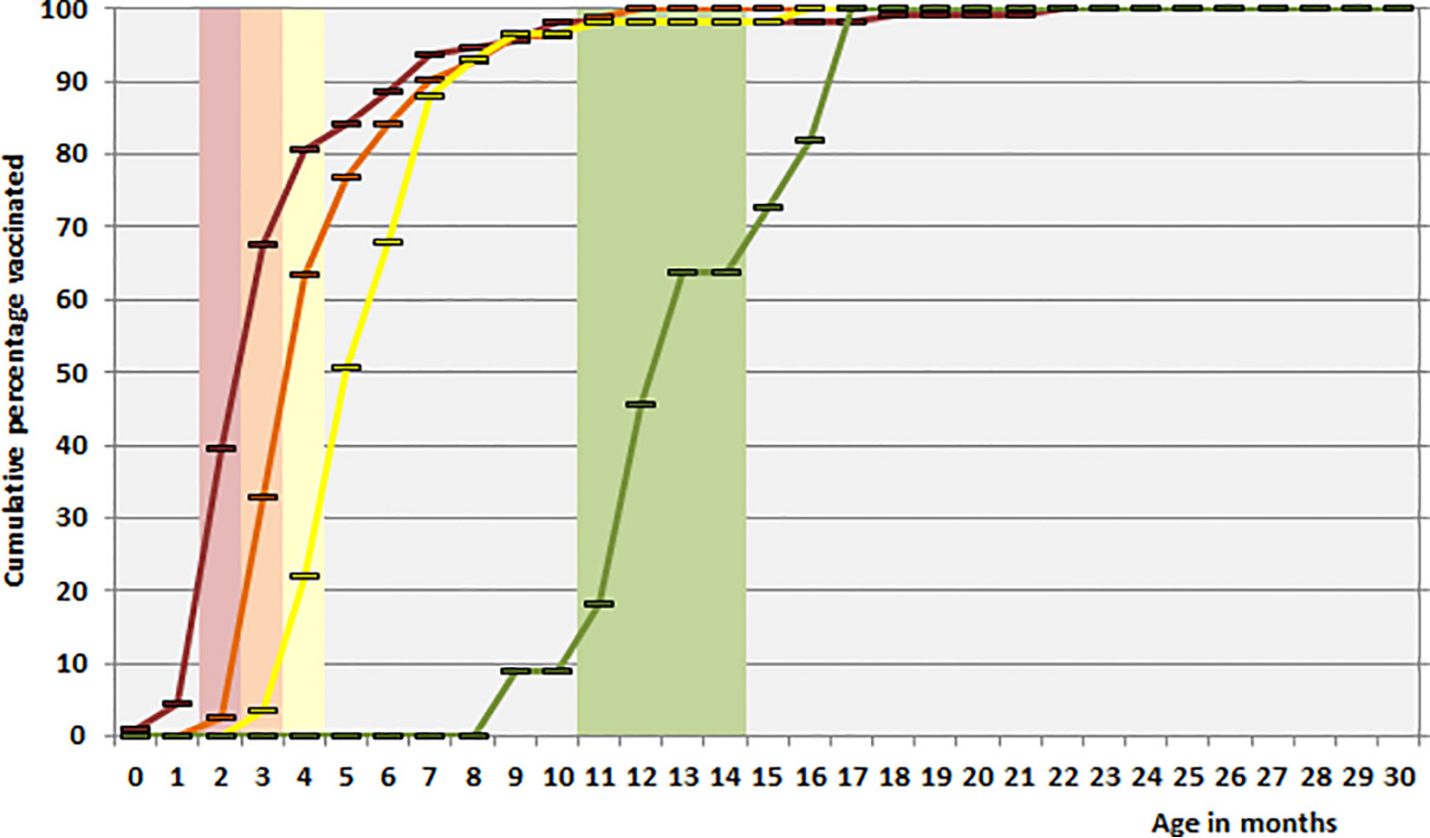
Timeliness of pneumococcal conjugate vaccination (PCV7) of children under two years of age with IPD in Germany (July 2006 –June 2010). Bars indicate scheduled vaccination times, lines indicate cumulative actual vaccination rates in percentage (red: 1^st^ dose, orange: 2^nd^ dose, yellow: 3^rd^ dose, green: booster dose). Only doses given before the time of infection were taken into consideration.

**Fig 3:**
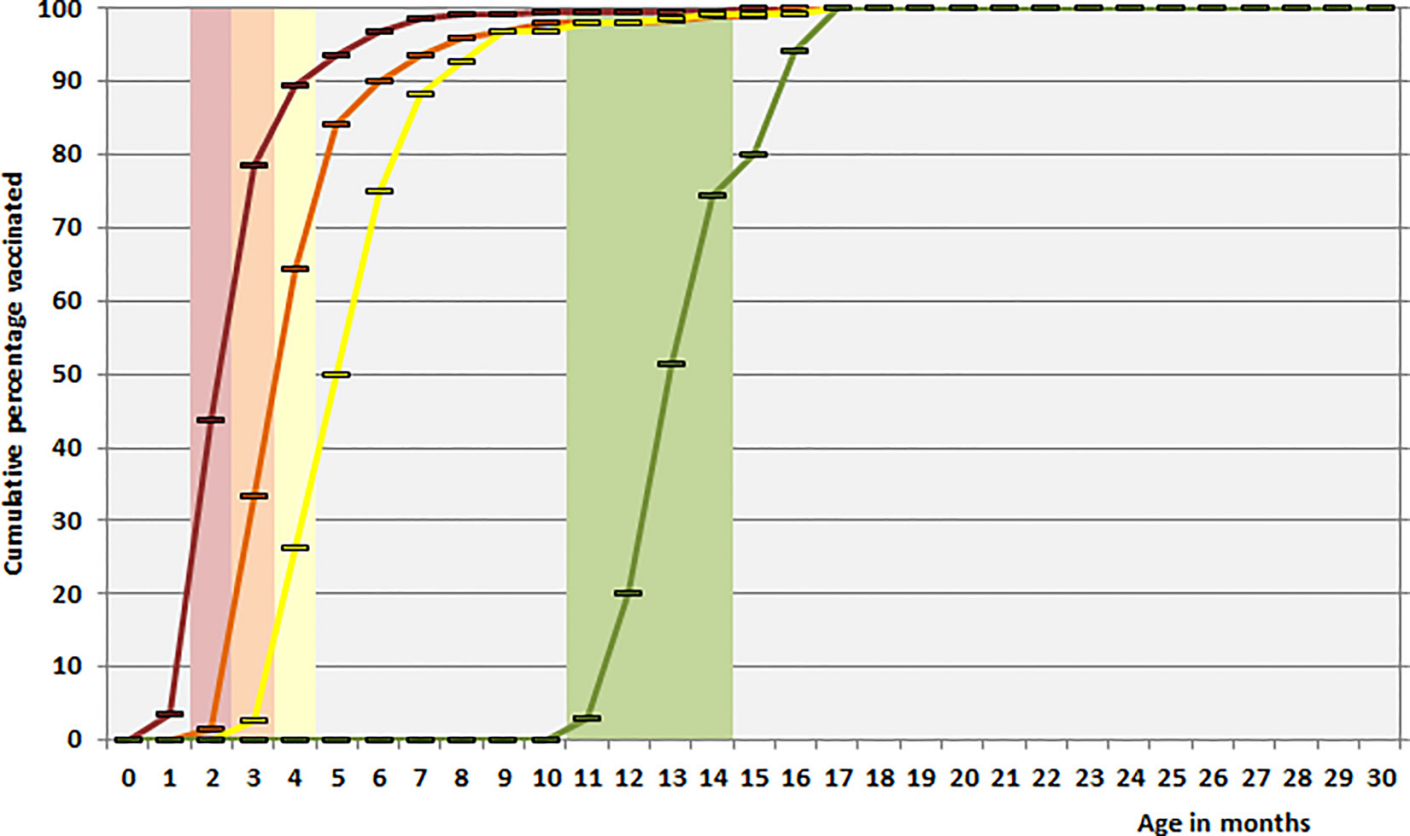
Timeliness of pneumococcal conjugate vaccination (PCV13) of children under two years of age with IPD in Germany (July 2010 –June 2015). Bars indicate scheduled vaccination times, lines indicate cumulative actual vaccination rates in percentage (red: 1^st^ dose, orange: 2^nd^ dose, yellow: 3^rd^ dose, green: booster dose). Only doses given before the time of infection were taken into consideration.

Among 114 children vaccinated with a first dose of PCV7 between July 2006 and June 2010, 45 (39.5%) had received this dose according to schedule (in the 3^rd^ month of life). Among 82 children vaccinated with a second dose, 27 (32.9%) had obtained their second dose in time, 13 of 59 children (22.0%) were vaccinated with a third dose according to schedule. Seven of 11 children (63.6%) had obtained their booster dose according to schedule (**Fig 2**). For children vaccinated with PCV13 between July 2010 and June 2015, proportions were somewhat higher: 43.8% (96/219), 33.5% (64/191), 26.3% (40/152) and 74.3% (26/35) respectively (**Fig 3**).

In the PCV7 period (July 2006 until June 2010), a total of 90 IPD cases caused by a serotype included in the vaccine occurred (**Table 3**). Of these, 73 (81.1%) were in unvaccinated children, and 15 (16.7%) in children that were incompletely vaccinated, i.e. that had not obtained the number of doses recommended for their age. Only two cases (2.2%) with PCV7 serotypes occurred in children vaccinated according to schedule.

**Table 3:** IPD cases among children under two years of age caused by serotypes included in PCV7 (2006–2010, among 141 non-vaccinated and 114 PCV7 vaccinated cases (A)) and PCV13 (2010–2015, among 98 non-vaccinated cases, 317 PCV13 vaccinated cases (B) and 11 PCV7 and 22 PCV10 vaccinated cases (C)).

	season	vaccination status	4	6B	9V	14	18C	19F	23F	1	3	5	6A	7F	19A	total
**A**	**2006–2007**	**not vaccinated**	1	11	2	13	3	5	7							**42**
		**incomplete (PCV7)**		1		2	2	2	1							**8**
		**according to age (PCV7)**					1									**1**
	**2007–2008**	**not vaccinated**		7	2	5	1	2								**17**
		**incomplete (PCV7)**		1		1	1	1	2							**6**
		**according to age (PCV7)**						1								**1**
	**2008–2009**	**not vaccinated**		1		4			1							**6**
		**incomplete (PCV7)**														**0**
		**according to age (PCV7)**														**0**
	**2009–2010**	**not vaccinated**		1		1	2	4								**8**
		**incomplete (PCV7)**						1								**1**
		**according to age (PCV7)**														**0**
	**2006–2010**	**all**	**1**	**22**	**4**	**26**	**10**	**16**	**11**							**90**
	** **	** **	** **	** **	** **	** **	** **	** **	** **							** **
**B**	**2010–2011**	**not vaccinated**		1			1	3		2	1			8	8	**24**
		**incomplete (PCV13)**								2	1				3	**6**
	**2011–2012**	**not vaccinated**		1				1			2		4	4	1	**13**
		**incomplete (PCV13)**									1			1	3	**5**
		**according to age (PCV13)**									1				1	**2**
	**2012–2013**	**not vaccinated**		2						1				1	2	**6**
		**incomplete (PCV13)**						1			2				4	**7**
		**according to age (PCV13)**													1	**1**
	**2013–2014**	**not vaccinated**						1		1	1			1	3	**7**
		**incomplete (PCV13)**						1			1				1	**3**
	**2014–2015**	**not vaccinated**				2				1	1				2	**6**
		**incomplete (PCV13)**													1	**1**
		**according to age (PCV13)**													1	**1**
	**2010–2015**	**all**		**4**	** **	**2**	**1**	**7**	** **	**7**	**11**	** **	**4**	**15**	**31**	**82**
**C**	**2010–2011**	**incomplete (PCV7)**													2	**2**
		**incomplete (PCV10)**													3	**3**
		**according to age (PCV7)**													2	**2**
		**according to age (PCV10)**		1												**1**
	**2011–2012**	**incomplete (PCV10)**				1										**1**
		**according to age (PCV10)**						1								**1**
	**2012–2013**	**incomplete (PCV10)**													1	**1**
** **	**2010–2015**	**all**		**1**	** **	**1**	** **	**1**	** **	** **	** **	** **	** **	** **	**8**	**11**

In the PCV13 vaccination period (July 2010 until June 2015), 82 IPD cases due to a vaccine serotype occurred (**Table 3**). Among these 56 (68.3%) were not vaccinated, 22 (26.8%) were incompletely vaccinated, and four (4.9%) were vaccinated according to age. As described above, 33 cases vaccinated with PCV7 and PCV10 were excluded from the PCV13 period (**Fig 1**). Interestingly, among these 33, another 11 cases with PCV13 serotypes were reported (**Table 3**).

In **Table 4** we have listed the residual vaccine serotype cases that occurred in vaccinated children. Both in the PCV7 period (2006–2010) as well as in the higher-valent vaccination period (2010–2015), the number of vaccine type cases decreased continuously. Most cases in the higher-valent vaccination period were serotype 19A (n = 23) but only three of these children were vaccinated according to schedule, and only one of those had obtained a full vaccination schedule of four doses.

**Table 4:** IPD cases among vaccinated children with PCV7 serotypes (2006–2010) and PCV13 serotypes (2010–2015).

Season	Diagnosis	Age (months)	Serotype	Vaccine	Vaccination status at infection
2006–2007	meningitis	10	6B	PCV7	no 2nd, no 3rd dose
	meningitis	4	14	PCV7	no 2nd dose
		3	14	PCV7	no 2nd dose
	meningitis	3	18C	PCV7	**according to schedule, 2 doses, infection four days after 2nd dose**
		4	18C	PCV7	no 2nd dose
	septic arthritis	4	18C	PCV7	no 2nd dose
	meningitis	12	19F	PCV7	no 2nd, no 3rd, no booster dose
		5	19F	PCV7	no 2nd dose
	meningitis	6	23F	PCV7	no 2nd, no 3rd dose
2007–2008	sepsis	3	6B	PCV7	no 2nd dose
		4	14	PCV7	no 2nd dose
	sepsis	3	18C	PCV7	no 2nd dose
		9	19F	PCV7	**according to schedule, 3 doses, infection at 286 days (9,5 months)**
	meningitis	13	19F	PCV7	no 3rd, no booster dose
		5	23F	PCV7	no 2nd dose
	meningitis	8	23F	PCV7	no 2nd, no 3rd dose
2009–2010	sepsis, otitis media	23	19F	PCV7	no booster dose
2010–2011	pneumonia	15	1	PCV13	no booster dose
	pneumonia	14	1	PCV13	no booster dose
	pneumonia	15	3	PCV13	no booster dose
	meningitis	12	6B	PCV10	**according to schedule, 4 doses**
		22	19A	PCV7	**according to schedule, 4 doses**
	otitis media, pharyngitis	10	19A	PCV10	no 3rd, no booster dose
		14	19A	PCV7	**according to schedule, 4 doses**
	pneumonia, sepsis, otitis media, mastoiditis	12	19A	PCV7	no booster dose
	phlegmon	15	19A	PCV13	no booster dose
	meningitis	17	19A	PCV10	no booster dose
	pneumonia	16	19A	PCV10	no booster dose
	pneumonia	20	19A	PCV7	no booster dose
	meningitis	11	19A	PCV13	no booster dose
	mastoiditis	11	19A	PCV13	no booster dose
2011–2012	pneumonia, pleuraempyema	15	3	PCV13	**according to schedule, 4 doses**
	pneumonia	15	3	PCV13	no 3rd, no booster dose
	meningitis, sepsis	5	7F	PCV13	no 3rd dose
	sepsis	18	14	PCV10	no booster dose
	sepsis	13	19A	PCV13	no booster dose
	sepsis, pneumonia, pleuraempyema	10	19A	PCV13	no booster dose
	mastoiditis	21	19A	PCV13	**according to schedule, 4 doses**
	mastoiditis	18	19A	PCV13	no booster dose
	mastoiditis	20	19F	PCV10	**according to schedule, 4 doses**
2012–2013	sepsis	10	3	PCV13	no booster dose
	HUS	19	3	PCV13	no booster dose
	meningitis, mastoiditis	13	19A	PCV13	no booster dose
	pneumonia, sepsis	3	19A	PCV13	**according to schedule, 1 dose**
	invasive myositis	14	19A	PCV10	no 3rd, no booster dose
	meningitis	4	19A	PCV13	no 2nd dose
	pneumonia	13	19A	PCV13	no booster dose
		5	19A	PCV13	no 2nd dose
	otitis media, mastoiditis	7	19F	PCV13	no 3rd dose
2013–2014	mastoiditis	17	3	PCV13	no booster dose
		14	19A	PCV13	no booster dose
	mastoiditis	10	19F	PCV13	no 2nd, no 3rd dose
2014–2015	pneumonia	22	19A	PCV13	no 3rd, no booster dose
	meningitis, sepsis	3	19A	PCV13	**according to schedule, IPD 10 days after first dose**

## Discussion

The vaccination data of 618 IPD cases among children under 2 years of age were available for our VE calculations. Our data show high VEs for both PCV7 and PCV13, which is in accordance with our data published on the changes in serotype distribution among both children and adults [4]. Adjusting for age and season following the issue of the vaccination recommendation resulted in slightly lower VE values in comparison to the crude values for most PCVs and single serotypes. Due to the quick disappearance of vaccine serotypes after start of vaccination, many VE estimates for single serotypes did not reach statistical significance.

The VE for PCV7 was high, reaching 97% after three doses, leading to a complete disappearance of vaccine type cases among vaccinated individuals who had received the full 3+1 schedule including the booster dose. Our VE estimate of 80% (95% CI: 63–89) for at least one dose was comparable to 79% (95% CI: 84–93) found in England and Wales [12], 90% (82–95) in Quebec [13], 88% (78–94) in the U.S.A. [14] and 91.3% (46.4–98.6) in Uruguay [15]. This shows little difference in effectiveness of a 3+1 schedule (Germany, USA) as compared to a 2+1 schedule (England and Wales, Quebec, Uruguay).

For the single serotypes, most VE values were comparable to those found in England and Wales [12]. For serotype 18C VE for at least one dose could not be demonstrated (8%; 95% CI: -239-76). This is in contrast with the adjusted value of 94% (95% CI: 64–99) found in England and Wales. However, the decrease from the unadjusted value of 56% was strongly driven by the fact that no 18C cases were received in the third post-PCV7 season (2008–2009). For comparison, 18C comprised 16.6% of isolates in 2006–2007, 5.4% in 2007–2008, and 4.8% of the isolates in 2009–2010. Also, due to the small number of cases, the CI of our VE estimate was very wide. None of the post booster VEs for single serotypes reached significance, because of the low number of cases. Our data suggest cross-protection towards serotype 6A, even though the VE values do not reach statistical significance.

The VE for PCV13 was high, reaching 91% (95% CI: 61–99) for all serotypes and 90% (54–98) for the six additional serotypes, post booster. The values of 75% (95% CI: 58–84; at least two doses before age 12 months or one dose on or after 12 months) and 79% (25–94) reported for a 2+1 dosing schedule in England, Wales and Northern Ireland, show similar effectiveness, with lower point estimates, but widely overlapping confidence intervals [16]. Harboe et al. report an 85% incidence reduction for the additional serotypes in PCV13 when comparing the PCV7 vaccination period with the PCV13 vaccination period [17] and Moore et al. report an incidence difference of 93% (91–94) between expected and actual case counts among children under 5 years of age in the USA, three years after the introduction of PCV13 [18].

Our data show effectiveness for at least one dose for serotypes 1 (83%; 95% CI: 15–97), 6A (96%; 56–100), 7F (84%; 18–98) and 19A (77%; 47–90), which is very similar to the effectiveness reported from England, Wales and Northern Ireland (84%; 54–95, 96%; 41–100, 91%; 70–98, 67%; 33–84) and Denmark (62%; -69-92, 88%; 64–96, no data on 6A and 19A). Our study shows an effectiveness of PCV13 for serotype 3 (74%; 95% CI: 2–93) for at least one dose of vaccine. This is in contrast to the VE (66%; 95% CI: -322-92) for two doses before age 12 months reported from England, Wales and Northern Ireland, and the VE (39%; 95% CI: -92-77) reported from Denmark where the point estimates are positive but the CIs are wide and include zero. The post primary and post booster point estimate VEs for serotype 3 in Germany are 80% (95% CI: -68-98) and 63% (95% CI: -393-97) respectively, but have CIs including zero, likely due to small cohort sizes. For the seven serotypes common to PCV7 and PCV13, a VE of 94% (95% CI: 78–99) for at least one dose was found, which is similar to the 80% (95% CI: 63–89) found for at least one dose of PCV7 and shows the high effectiveness of PCV13 towards these serotypes.

Our data on the timeliness of pneumococcal conjugate vaccination among children with IPD show a considerable delay in the administration of the doses. One fifth of the children had not had a single dose of PCV7 at age 4 months, for PCV13 this was 11%. And only 22.0% (PCV7) and 26.3% (PCV13) of children finished their primary schedule in time. Booster doses are also delayed, with only two thirds (PCV7) and three quarters (PCV13) of the children being boostered within the scheduled time. It should be noted that we only analysed data from children with IPD and that these children may not be representative for all children in Germany. Interestingly enough, when we calculated the timeliness of vaccination using only non-vaccine type IPD, the values were only marginally different (0–1%). Furthermore, the Robert Koch Institute has published data on the timeliness of pneumococcal vaccination among healthy children showing a very similar delay in administration of the doses, with only 40–50% of children having a had their third dose at age of 6 months and only 30–40% having had a booster dose at age 15 months [19]. The delays in administration of PCVs in Germany may in part be due to the fact that STIKO only issues recommendations, but vaccinations are given by pediatricians working in their own practices and there is no incentive for either parents or pediatricians to adhere to the recommended schedules. The delay results in many children having insufficient protection, and may have been one of the reasons the children in our cohort got IPD. A vaccination uptake of >90% is only reached with a 3–5 month delay for the primary doses, and not until the age of 17 months have all children received a complete 3+1 schedule. In this light, the recent change (August 2015) of the STIKO recommendation from a 3+1 to a 2+1 schedule, leaving out the second dose at 3 months of age [19, 20], has to be critically assessed. Data from England,Wales and Northern Ireland, Norway and Denmark show the high effectiveness of 2+1 schedules. However, vaccination uptake in these countries exceeds 90% [17, 21, 22] and in England there are incentives for practicioners encouraging strict adherence to vaccination schedules [23, 24]. If the 2+1 schedule is implemented in Germany with similarly poor adherence, this may result in a diminished level of individual as well as herd protection.

Exemplifying the lack of protection due to delayed administration are the vaccine type cases reported during the PCV7 and PCV13 vaccination periods. Of all cases with a PCV7 serotype, 81.1% were not vaccinated and 16.7% were incompletely vaccinated, and for a large part this was due to a delayed administration of primary doses. There were no specific predominant vaccine serotypes in these incompletely vaccinated cases, however, the only three cases with three primary doses but lacking the booster dose were all 19F. We have reported on a case of 19F IPD in a non-boostered child before [25] and it seems that 19F is persisting in the community longer than other vaccine serotypes as published recently by our group [4].

In the PCV13 period, 26.8% (n = 22) of IPD cases with a PCV13 serotype occurred in children incompletely vaccinated with PCV13. Seventeen cases lacked a booster dose and ten of them were serotype 19A. Another five cases with serotype 19A were found among children vaccinated with PCV10 and lacking a booster dose. This once more shows the importance of the booster dose, but also the persisting circulation of serotype 19A among children in the PCV13 vaccination period, similar to the persistence of serotype 19F in the PCV7 period [4].

Our study has several limitations. Aside from systemic immunity, pneumococcal conjugate vaccines also illicit mucosal immunity. Therefore, they reduce the carriage of vaccine serotypes in immunized children, and thus reduce the circulation of vaccine serotypes among children. This means that even non-vaccinated children have a lower chance of carrying and getting infected with vaccine serotypes (herd protection), albeit with a temporal delay compared to the vaccinated children. Andrews et al. [12, 16] have assessed this bias by correcting for the difference in probability of non-vaccine type carriage in both vaccinated and unvaccinated individuals. Since data on carriage are not available for Germany, we could not use this correction method and our vaccine effectiveness data are likely to be slightly overestimated. Adjusting our data with the carriage parameters used by Andrews et al. showed an overestimation of 0.5–5% for most VE values. Furthermore, our data were not adjusted for underlying disease or other risk factors, because these data were not available as IPD is not a notifiable disease in Germany, and as such pertinent information on our case report forms was limited. Another limitation to our study, and indeed in many other published studies using logistic regression to estimate odds ratios for VE calculations, is that low event numbers (at least 5–10 events per predictor variable are recommended [26, 27]), which commonly occur in the rapidly-disappearing vaccine-type serotypes, strongly contribute to instability in the results of the regression models, and thus the VE calculations of these small cohorts should not be strictly interpreted, as values could be misleading. This is particularly evident in the PCV7 post booster VEs for serotypes 4 and 9V, where case numbers are so low that adjustment results in negative point estimates and extremely wide CIs.

## Conclusions

Our data show a high vaccine effectiveness for both 7- and 13-valent conjugate vaccines used in Germany administered in a 3+1 schedule. For the individual serotypes, the calculated effectiveness was high both for the serotypes common to PCV7 and PCV13 as well as for the additional serotypes in PCV13, including serotype 3. Most vaccine type IPD cases occurred in unvaccinated or only partially vaccinated children. Better adherence to the recommended vaccination schedule could have prevented most of these cases. Careful monitoring of the effects of the recent change from a 3+1 schedule to a 2+1 schedule on the incidence of vaccine type pneumococcal disease in Germany is necessary.
